# X chromosome transmission ratio distortion in *Cftr *+/- intercross-derived mice

**DOI:** 10.1186/1471-2156-8-23

**Published:** 2007-05-16

**Authors:** Christina K Haston, Daryl G Humes, Melanie Lafleur

**Affiliations:** 1 Meakins-Christie Laboratories and Department of Medicine, McGill University, 3626 St. Urbain, Montreal, Quebec H2X 2P2, Canada

## Abstract

**Background:**

Cystic fibrosis (CF) mice, created with a genetically engineered mutation in the Cystic fibrosis transmembrane conductance regulator (*Cftr*) gene, may develop intestinal plugs which limit their survival past weaning. In a studied population of genetically mixed CF mice differences in allelic ratios at particular loci, between surviving CF mice and mice with the lethal intestinal defect, were used to map cystic fibrosis modifier gene one, *Cfm1*. Using this approach, we previously identified an X chromosome locus which may influence the survival to weaning of C57BL/6J × BALB/cJ F2 CF mice. We also detected two regions of transmission ratio distortion, independent of *Cftr *genotype, in a limited dataset. To investigate these findings, in this study we have genotyped 1208 three-week old F2 mice, and 186 day E15.5 embryos, derived from a congenic (C57BL/6J × BALB/cJ) F1 *Cftr *+/- intercross, for the putative distortion regions.

**Results:**

An excess of homozygous BALB genotypes, compared to Mendelian expectations, was detected on chromosomes 5 (p = 5.7 × 10^-15^) and X (p = 3.0 × 10^-35^) in three-week old female mice but transmission ratio distortion was not evident in the tested region of chromosome 3 (p = 0.39). Significant pre-weaning lethality of CF mice occurred as 11.3% (137/1208) of the three-week old offspring were identified as CF mice. X chromosome genotypes were not, however, distorted in the female CF mice (p = 0.62), thus the significant non-Mendelian inheritance of this locus was dependent on CF status. The survival of CF embryos to day E15.5 was consistent with Mendelian expectations (42/186 = 23%), demonstrating the loss of CF mice to have occurred between E15.5 and three weeks of age. The excess of X chromosome homozygous BALB genotypes was recorded in female embryos (p = 0.0048), including CF embryos, indicating the distortion to be evident at this age.

**Conclusion:**

Two of three previously suggested loci of transmission ratio distortion were replicated as distorted in this mouse cross. The non-Mendelian inheritance of X chromosome genotypes implicates this region in the survival to weaning of non-CF mice.

## Background

One of the clinical manifestations of cystic fibrosis (CF) not adequately predicted by the cystic fibrosis transmembrane conductance regulator (CFTR) genotype is the intestinal phenotype meconium ileus [1]. *Cftr*-knockout mouse models show intestinal plugging at birth, resembling the clinical phenotype, and the strain-dependent incidence of this lethal complication has been used to map a genetic modifier of CF intestinal disease, named cystic fibrosis modifier gene 1 [2].

We have mapped potential modifiers of the CF intestinal phenotype [3] in C57BL/6J (B6) × BALB F2 CF mice, using an approach based on genotypic differences between surviving CF mice and mice succumbing to intestinal disease. In that study, the genetic influence on CF mouse survival was demonstrated by the fact that more F2 CF mice were alive at weaning (54% of the expected number of CF mice lived to this time point) than were congenic B6 (22%) or BALB (13%) CF mice. We identified an X chromosome locus which may contribute to this survival difference, in that non-Mendelian inheritance favoring B6 alleles in the surviving CF mice and BALB alleles in mice of a control population, was observed. Two loci of transmission ratio distortion, independent of *Cftr *genotype, on chromosomes 3 & 5, were also detected in the female mice of this cross. Based on birth records the CF mice not surviving to weaning were presumed to have succumbed during both the prenatal and postnatal periods. We therefore concluded the phenotype of survival to weaning to be from either death due to intestinal distress in the perinatal period as in [2,4], or from death *in utero*, which may not be due to intestinal distress specifically.

As in our previous study [3], the putative loci of transmission ratio distortion were identified in 96 control mice, in this report we typed mice of a larger B6 × BALB F2 cohort (n = 1208) to investigate the CF dependence of the potential X chromosome modifier locus, and to confirm or refute the non-Mendelian inheritance of genotypes at loci on chromosomes 3 & 5. In addition, to narrow the time frame of the influence of the potential cystic fibrosis modifier we evaluated embryonic mice of the same cross.

## Results

To investigate whether previously identified regions of transmission ratio distortion (TRD) existed in a larger cohort of B6 × BALB F2 mice, we bred 1208 mice and genotyped the population for markers on chromosomes 3, 5, and X. As shown in Table 1, two of the loci of TRD persisted as the female mice of this cross had an excess of the homozygous BALB genotype at the tested markers on chromosomes 5 and X while the distortion was not evident in male mice nor at the chromosome 3 marker.

To determine whether the confirmed transmission ratio distortion of the chromosome 5 and X genotypes was dependent on *Cftr *genotype, we next typed the three-week old F2 mice for CF status (CF or non CF). Of the 1208 mice evaluated 137 were identified to be CF and this number is fewer than would be expected for an autosomal recessive trait (25% of the population sample of 1208 mice = 302 mice expected). We therefore conclude that pre-weaning lethality of CF mice occurred. The *DXMit16 *genotypes for the subset of our population which were identified to be CF mice were evaluated, and as shown in Table 1, X chromosome genotypes were not distorted from Mendelian expectations in this sample of F2 CF female and male mice. We next compared the set of *DXMit16 *genotypes of CF mice to that of non-CF littermates and observed a lack of genotype distortion in female CF mice. This makes the CF dataset distinct from that of female non-CF mice for *DXMit16 *genotypes (p = 8.7 × 10^-6^), due to the non-Mendelian inheritance of genotypes for this region in the latter group. To further investigate the CF dependence of the putative distortion, we grouped the non-CF female F2 mice by *Cftr *+/+ and +/- genotype and found the *DXMit16 *genotype to be independent of *Cftr *genotype (p = 0.12) in control mice. The genotypes at marker *D5Mit239 *of B6 × BALB F2 CF female mice were also not distorted from Mendelian expectations, as shown in Table 1, although an excess of BALB alleles may be present in this population (p = 0.06). The set of *D5Mit239 *genotypes of the female F2 CF mice is not different from those of the non-CF mice (p = 0.26), therefore the distortion at this marker is independent of *Cftr *genotype which is in keeping with our first report.

**Table 1:** Genotype frequency in 3 week old B6 × BALB F2 CF and non-CF mice

	**Marker**	**Position (Mb)^a^**	**No. mice**	**No. B6:B6**	**No. B6:BALB**	**No. BALB:BALB**	**P-value^b^**
**F2 Non-CF 3 weeks**							
*Females*	*D5Mit239*	107.7	707	145	292	270	5.7 × 10^-15^
	*DXMit16*	95.6	687	210	188	289	3.0 × 10^-35^
	*D3Mit189*	101.1	367	101	171	95	0.39
*Males*	*D5Mit239*	107.7	358	74	192	92	0.16
	*DXMit16*	95.6	341	154^c^	0	187	0.07
	*D3Mit189*	101.1	364	98	176	90	0.69
**F2 CF 3 weeks**							
*Females*	*D5Mit239*	107.7	58	9	30	19	0.17
	*DXMit16*	95.6	56	14	31	11	0.62
	*D3Mit189*	101.1	54	16	23	15	0.54
*Males*	*D5Mit239*	107.7	75	9	45	20	0.035
	*DXMit16*	95.6	79	35	0	43	0.37
	*D3Mit189*	101.1	75	15	40	20	0.61

^a^taken from the Ensembl database, release 40, August 2006.

^b^P-values obtained by standard Pearson's χ^2 ^test comparing observed values to expected 1:2:1 ratio (B6/B6:B6/BALB:BALB/BALB) of genotypes (except for 1:1 X chromosome genotypes of male mice). B6 = C57BL/6J, BALB = BALB/cJ.

^c ^*DXMit16 *genotype is B6 or BALB in male mice.

The distortion of genotypes at *DXMit16 *in female mice of the current study is consistent with what we observed in a previous cross of the B6 and BALB strains [3], as shown in Table 2, while the genotypes of the additional F2 CF males do not support the trend identified in the previous dataset. In the prior study, F2 CF male mice were found to have an excess of B6 alleles at *DXMit16*, and, as such distortion is not evident in this dataset, the original finding may have been due to insufficient sample size. The previous study also differs from the current work in that, previously, BALB *Cftr *+/- mice were crossed with B6 *Cftr *+/- mice to generate the F1 mice while in the current study the BALB parents were *Cftr *+/+. This change results in the CF mutation being solely inherited from the B6 chromosome in the present study. The consequence of this change would be the non-Mendelian inheritance of genotypes (favouring B6 alleles) in the chromosome 6 region of *Cftr *in the CF mice of this cross. As the change in breeding strategy would not affect the inheritance of alleles on other chromosomes, we combined the datasets of the two investigations for further analysis. Comparing the genotypes of the total non-CF mice of both studies, to the combined genotypes of CF mice, reveals a significant difference in *DXMit16 *genotype ratios, by CF status, in both male (p = 9.0 × 10^-4^) and female mice (p = 3.4 × 10^-16^). From these data we conclude the non-Mendelian inheritance of the X chromosome marker in F2 mice of a B6 × BALB *Cftr *+/- intercross to depend on CF status.

**Table 2:** *DXMit16 *Genotype frequency in 3 week old CF and non-CF mice of replicate studies

	**dataset**	**No. mice**	**No. B6:B6**	**No. B6:BALB**	**No. BALB:BALB**	**P-value^c^**
**F2 Non-CF 3 weeks**						
*Females*	Present^a^	687	210	188	289	3.0 × 10^-35^
	HSC^b^	45	5	23	17	0.04
	total	732	215	211	306	3.8 × 10^-34^
*Males*	Present	341	154^d^	0	187	0.05
	HSC	51	17	0	34	7.6 × 10^-4^
	total	392	171	0	221	0.012
**F2 CF 3 weeks**						
*Females*	Present	56	14	31	11	0.62
	HSC	134	32	75	27	0.32
	total	190	46	106	38	0.20
*Males*	Present	79	34	0	45	0.22
	HSC	126	79	0	47	5.5 × 10^-5^
	total	205	113	0	92	0.14

^a^Present data set; ^b^Data taken from Haston & Tsui 2003, from the Hospital for Sick Children.

^c^P-values obtained by standard Pearson's χ^2 ^test comparing observed values to expected 1:2:1 ratio (B6/B6:B6/BALB:BALB/BALB) of genotypes, (except for 1:1 X chromosome genotypes of male mice). B6 = C57BL/6J, BALB = BALB/cJ.

^d^*DXMit16 *genotype is B6 or BALB in male mice.

Our analyses also confirmed the significant incidence of pre-weaning lethality in B6 × BALB F2 CF mice (137/302 = 45% of the expected number of CF mice were identified at weaning). To narrow the time frame in which this lethality occurred, we assayed day E15.5 embryos of nineteen litters and determined CF embryo survival at this age. The *Cftr *genotypes of the embryos were 40:104:42 (*Cftr *+/+: +/-: -/-) which is consistent with Mendelian expectations (p = 0.27) and which also indicates that the lethality of the CF mice of this cross occurred between embryonic day 15.5 and the age of three weeks. Finally, to further investigate the potential transmission distortion of markers *D5Mit239 *and *DXMit16 *in the mice of this cross, the embryos were typed for these loci and for a Y chromosome marker to indicate their sex. At marker *D5Mit239 *the inheritance of B6/BALB genotypes was consistent with Mendelian expectations in both female (p = 0.41) and male (p = 0.35) embryos, data not shown. For marker *DXMit16 *the transmission ratio distortion identified in female mice at weaning was evident in the embryos, as the genotypes of the females were (35 BALB/BALB: 44 BALB/B6: 13 B6/B6, p = 0.0048, compared to an expected ratio of 1:2:1) and of males were (51 BALB: 37 B6, p = 0.14, compared to an expected ratio of 1:1). The genotype ratios for the subset of embryos identified as CF were consistent with those reported for the larger group (9:14:1 in CF embryonic females and 11:4 in males).

## Discussion

Herein we confirm the existence of transmission ratio distortion at specific loci on chromosomes 5 and X in the female mice of a B6 × BALB *Cftr *+/- intercross. In this replicate study we also show the non-Mendelian inheritance of X chromosome genotypes to influence the survival to weaning of *Cftr *+/+ and *Cftr *+/- mice, but not *Cftr *-/- mice, which suggests the mechanism leading to TRD in this cross may involve functional Cftr.

With the completion of this second study, our observation of X chromosome transmission ratio distortion as influenced by the CF status of the mice is now based on genotypes of 395 CF mice and 1184 non-CF mice, compared to the 96 non-CF mice evaluated in the original report. These numbers of mice are in line with those used to identify X chromosome transmission ratio distortion, or non-Mendelian inheritance, in offspring of different mouse crosses [5,6] and, further, the marker of distortion we report on maps to one of these previously defined intervals. We also provide evidence for a second locus of non-Mendelian inheritance, in this cross, on chromosome 5 which affected females only, and we illustrate the importance of sufficient sampling in transmission ratio distortion studies as no distortion of the locus on chromosome 3, now tested in 367 females compared with 45 in our prior study [3] was evident.

The previous studies of transmission ratio distortion have attributed distortion to prenatal lethality associated with particular allelic combinations but the causative genes producing the non-Mendelian inheritance have not been uncovered. Our embryonic data supports a prenatal effect as the distortion of *DXMit16 *genotypes was evident at day E15.5. This effect did not, however, influence the survival of CF embryos to E15.5, which was consistent with Mendelian expectations. As the survival of CF mice to weaning was clearly below expectations (45% of the expected number of one fourth of the litters), and as the strong distortion of *DXMit16 *genotypes, evident in control mice, was absent in CF mice, the mechanisms leading to these observations may be linked and likely act in mice between the ages of E15.5 and 3 weeks. Further study is needed to identify the X-linked modifier which may enhance the survival of CF mice in the perinatal period.

The survival advantage conferred to control mice by inheritance of the X chromosome homozygous BALB genotype was not evident in CF mice of replicate populations thus, the mechanism leading to transmission ratio distortion in the mice of this cross may involve Cftr protein function. One potential explanation for this may be in Cftr expression as Larson *et al*. [7] have shown *Cftr *gene therapy *in utero *to lead to death of *Cftr *+/+ embryos, while *Cftr *-/- embryos were spared. The F2 non-CF mice that inherited B6/BALB heterozygous genotypes on chromosome X may have expressed a level of *Cftr *that is incompatible with survival to weaning. X chromosome candidate genes for such an interaction can be taken from protein complexes reported to regulate Cftr, although its regulation is not completely understood [8]. This list includes signalling molecules, kinases, transport proteins, PDZ-domain-containing proteins, myosin motors, Rab GTPases, and SNAREs [8], as does the distorted region of the X chromosome. For example, transcription factors, such as Aristaless-related homeobox gene, *Arx*, which has a crucial role in pancreas development [9], and *Dax-1 *which is involved in epithelial differentiation [10], map to the distorted region on X and could influence Cftr-affected pathways. One such pathway, for which mutations in both *Dax-1 *[11] and *Cftr *[12] have been implicated, is male infertility. Elucidation of an X chromosome factor which influences Cftr activity, if such exists, could be an important advance in understanding cystic fibrosis disease development.

## Conclusion

We have provided evidence for two loci of transmission ratio distortion in the female mice of a B6 × BALB F2 intercross. One locus, on the X chromosome, was shown to have non-Mendelian inheritance in the non-CF mice of this cross, but not to be distorted in the in the subset of mice identified to be CF. The CF status of the mouse may therefore be sparing the effect produced by the distorted inheritance of the X chromosome. Such an interaction, if confirmed, could provide important information on *Cftr *regulation in the developing mouse.

## Methods

### Mice

The mice of the C57BL/6 *Cftr*+/tm1UNC (*Cftr*^+/-^) strain were provided by Dr. Danuta Radzioch of the Montreal General Hospital, and the BALBc/J (BALB) strain was purchased from the Jackson Laboratory. F2 CF mice were created from these strains through two generations of breeding. Specifically, the B6 *Cftr*^+/- ^and BALB mice were crossed to create F1 mice and the resultant F1 *Cftr*^+/- ^mice were intercrossed to create 1208 F2 non-CF (*Cftr*^+/+ ^and *Cftr*^+/-^) and CF (*Cftr*^-/-^) mice which were identified by their *Cftr *genotypes at weaning (~3 weeks of age). All mice were housed in micro-isolator cages in a Specific Pathogen Free room and handled according to the standard husbandry of the Animal Resource Centre of McGill University. Animal use and handling was approved by the McGill University Committee for Animal Care and Use, which follows the guidelines and regulations of the Canadian Council on Animal Care.

### Embryos

F1 *Cftr *+/- mice were intercrossed and 186 embryos were dissected from the uteri of 19 sacrificed females on day E15.5. Embryonic day 15.5 was chosen as individual embryos are of sufficient size for genotyping at this time, and as data from Bonner *et al*. [13] show the embryonic expression of *Cftr*, in the lungs, to have reached a plateau at this age. At sacrifice the amniotic sac from each intact embryo was removed and used as a source of genomic DNA.

### Genotyping

DNA was prepared from the tail clips of the three week-old F2 mice and from the amniotic sacs of the F2 embryos. Polymorphic mouse genetic markers, described by Dietrich *et al*. [14], were amplified by PCR for the genotyping of three specific loci on chromosomes 3, 5 and X (see Table 1). The PCR products were separated by electrophoresis on 3% agarose gels and visualized with ethidium bromide staining. The sex of the embryos was determined by amplifying and detecting a portion of the *Sry1 *gene, a Y chromosome-specific marker, as in [15]. The *Cftr *genotypes of the three week-old F2 mice and of the E15.5 embryos were determined by performing a previously described PCR assay [16] using the isolated genomic DNA.

### Inheritance of B6 and BALB alleles

Deviations in genotypic ratios compared to the expected Mendelian ratio of 1:2:1 (B6/B6:B6/BALB:BALB/BALB) and in allelic ratios compared to the expected 1:1 ratio (B6 alleles: BALB alleles), were assessed by χ^2 ^analysis (as in [6]) using Microsoft Excel software.

## Authors' contributions

CKH conceived of the study, completed the analyses and wrote the manuscript. DGH genotyped the mouse cohort, and ML maintained the animal breeding and completed the embryonic experiment. All authors read and approved the final manuscript.
